# Role of hybrid nanofiller GNPs/Al_2_O_3_ on enhancing the mechanical and tribological performance of HDPE composite

**DOI:** 10.1038/s41598-023-39172-9

**Published:** 2023-08-01

**Authors:** Nabhan A., Mohamed Taha, Ahmed Mohamed Mahmoud Ibrahim, Ameer A. K.

**Affiliations:** 1 Production Engineering and Mechanical Design Department, Faculty of Engineering, Minia University, El-Minia, 61519 Egypt; 2 Mechanical Engineering Department, College of Engineering and Technology, Arab Academy of Science, Technology and Maritime Transport, Sadat Road, P.O. Box 11, Aswan, Egypt

**Keywords:** Mechanical engineering, Nanoscale materials, Structural materials

## Abstract

The unique mechanical properties and wear resistance of HDPE give it the potential as an alternative to frictional material. The current research focuses on using hybrid nanoparticles with various loading fillers to determine the best additive contents. The mechanical and tribological characteristics were examined and evaluated. The HDPE nanocomposite samples containing 0.5, 1.0, 1.5, and 2.0 wt.% filling content of Al_2_O_3_ nanoparticles (NPs) and 0.5, and 1.0 wt.% of graphene nanoplatelets (GNPs) were fabricated. The results showed a good enhancement in the mechanical and tribological properties of HDPE composites with the presence of nano additives. The HDPE nanocomposites recorded the best performance with a loading amount of 2.0 wt.% with an equal ratio of hybrid nanofiller Al_2_O_3_ NPs and GNPs.

## Introduction

Polyethylene (PE) is one of the most popular polymers with the uniqueness of being very useful in a wide range of fields other than its cost-effectiveness. High-density polyethylene HDPE is produced at appropriate temperatures and pressures to control the formation profile. The target chain of HDPE is formed in a linear structure with a few slight branching^1^. Many nanomaterials are adopted as fillers for polyethylene to enhance the chemical bonding of the polymer structure. Many nano-additives such as carbon nanofibers, carbon nanotubes, and Al_2_O_3_ nanoparticles were performed to evaluate the wear resistance and friction coefficient of PE nanocomposites^2^. Both high-density polyethylene (HDPE) and Ultra high molecular-weight polyethylene (UHMWPE) are widely used as bearing materials in many industries. This is due to the fact that it has an outstanding resistance property and low effective costs^3^.

In many fields such as friction materials in the automotive industry^4,5^, pressure pipes^6^, and low-speed bearings^7^, it is mainly based on HDPE. On the other hand, industries like artificial joints and wear strips adopt UHMWPE as the base material^8,9^. UHMWPE nanocomposite was successfully fabricated by adding an eco-friendly additive (rice straw cellulose nanofibers) resulting in preferable mechanical strength, depressed friction coefficient, and wear rate^10^. Recently, UHMWPE is no longer used only in hip replacements but is also a base material for overall knee replacements^11,12^. The numerical model was constructed using ANSYS and MATLAB to evaluate the wear of the hip replacement^13^. In the field of artificial hips, HDPE has been modified to perform a high wear resistance and low rubbing forces^14,15^. Furthermore, UHMWPE achieves wear rates as low as 0.25 mm/year, which helps to take control of artificial hip problems^9^.

In recent decades, the world has tended to develop and improve the properties of polymers. This led to that nanofillers have a distinguished role in enhancing wear resistance and material strength^16–19^. Several studies have been done to enhance the tribological and mechanical properties of HDPE by incorporating it with different nanomaterials, such as carbon nanotubes (CNT)^20,21^, or graphene oxide^22–24^. Al_2_O_3_ nanoparticles are a superb filler element in the PE matrix since it is low cost and it helps to progress the tribological and mechanical performance of the composite^5,25^. It was reported that the low loading content of Al_2_O_3_ NPs was applied as a filler to improve the tribological performance. The HDPE was reinforced with 0.1. 0.2, 0.3, 0.4, and 0.5 wt.% of Al_2_O_3_ NPs. The nanocomposites exhibit a good reaction because of adding Al_2_O_3_ NPs. Moreover, the coefficient of friction was reduced by 11%, while the hardness increased by 9.1%^5^. The incorporation of Al_2_O_3_ NPs into UHMWPE plays a vital role in inhibiting oxidation and enhancing wear resistance^26^. While the stiffness, resistance of crack growth, and high impact resistance were recorded as clearly enhanced for HDPE reinforced with CaCO_3_ NPs^27^. MWCNTs are considered a typical filler to polymeric matrix, which plays a noticeable role in modifying the mechanical and tribological properties and contributes to the rise of the service life^28,29^. The compositions of 0.2–2.0 wt.% of MWCNTs were adopted as a filler material of HDPE. The results revealed that the wear rate of the composites decreases with increasing of MWCNTs content^20^. Moreover, nano-diamond ND, MWCNTs, and graphene nanoplatelets GNPs were added to modify the friction performance of HDPE-based nano-composites. The HDPE/ND composites exhibit a distinct wear performance in the comparison with the pure HDPE^30^. It can be concluded that the bonding reaction between the filler and the HDPE matrix leads to improving shear modulus and wear resistance. MWCNTs/HDPE nanocomposites were adopted with loading content of 0.5, 1.0, 1.5, 2.0, and 2.5 wt.%. The mechanical properties of nanocomposites increase with increasing the loading content up to 2.0 wt.%. MWCNTs/HDPE nanocomposite with composition of 2.0 wt.% exhibited the best tribological performance^31^.

The hybrid filler has an extra feature where it is possible to achieve an extra benefit from both materials, which contributes to enhancing the properties of the composites. The hybrid nanofiller GNPs and polyaniline (PANI) were performed to prepare HDPE composites. The loading content of filler was 10 wt.% with a 1:4 ratio of GNPs/PANI. The results evidence that the HDPE nanocomposite exhibits distinct electrical conductivity and low surface and volume resistivity^32^. Also, the HDPE matrix reinforced by Carbon nanofibers (CNFs) with silane coatings was fabricated and the tribological performance has been evaluated. The loading amount of the hybrid nanofiller was 0.5%. 1.0% and 3 wt.%. It can be indicated that the low filling amount has an excellent influence on the tribological performance, where the wear rate was reduced by about 35% at 0.5 wt.% of the loading amount. Additionally, it was observed that the higher contents of nano additives increase the wear rate, which may be attributed to the presence of aggregates of the filler^33^. Mechanical and tribological properties of HDPE reinforced by Alumina-toughened Zirconia (ATZ) with compositions 2–12 wt.%. It can be indicated that the modulus of elasticity and strength was enhanced due to good dispersion and low agglomerations. Moreover, the nanocomposites exhibit a distinct wear resistance^34^.

The scope of the work consists of studying the possible enhancing effect of hybrid additives of Al_2_O_3_ NPs and GNPs as nanofiller for HDPE matrix. The loading content of 0.5 wt.%, 1.0 wt.%, 1.5 wt.%, and 2.0 wt.% of Al_2_O_3_ NPs and 0.5%, and 1.0 wt.% of GNPs were added. The mechanical and tribological properties of HDPE nanocomposites were evaluated in the comparison with pure HDPE.

## Experimental details

### Materials and methods

**Figure 1:**
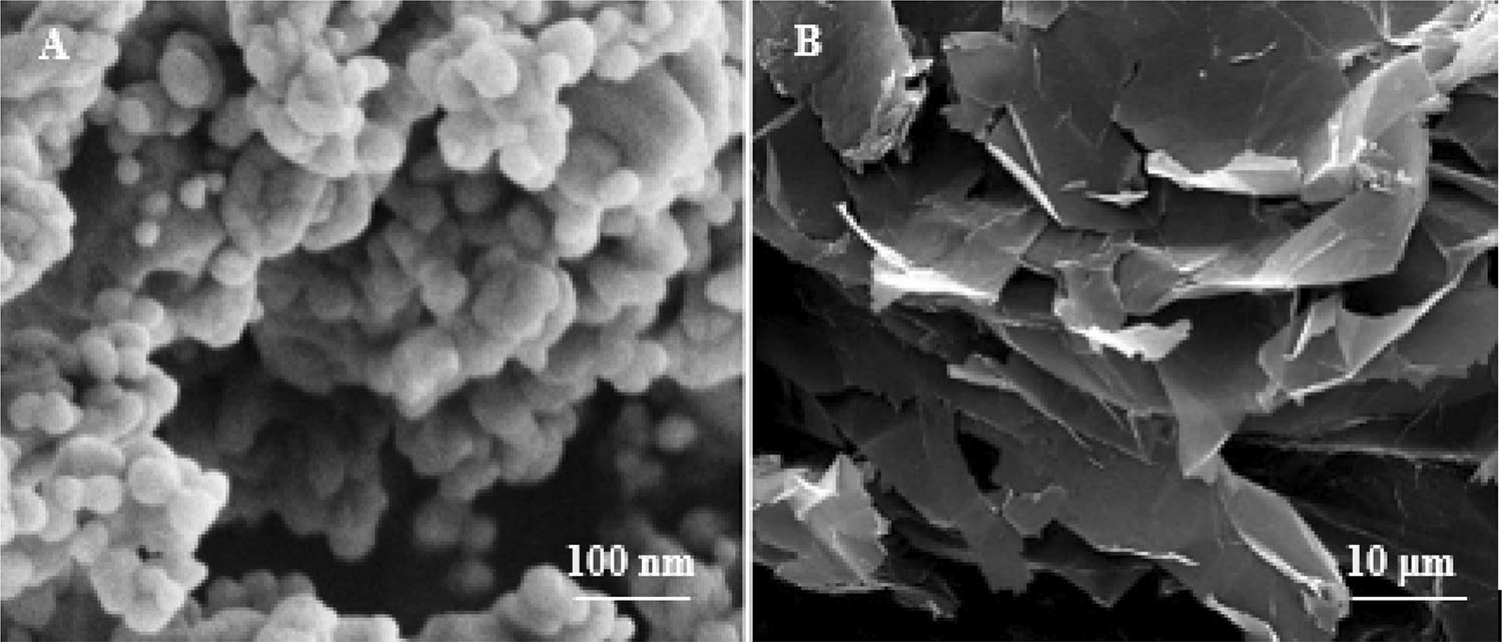
SEM images of Al_2_O_3_ NPs and GNPs.

**Figure 2:**
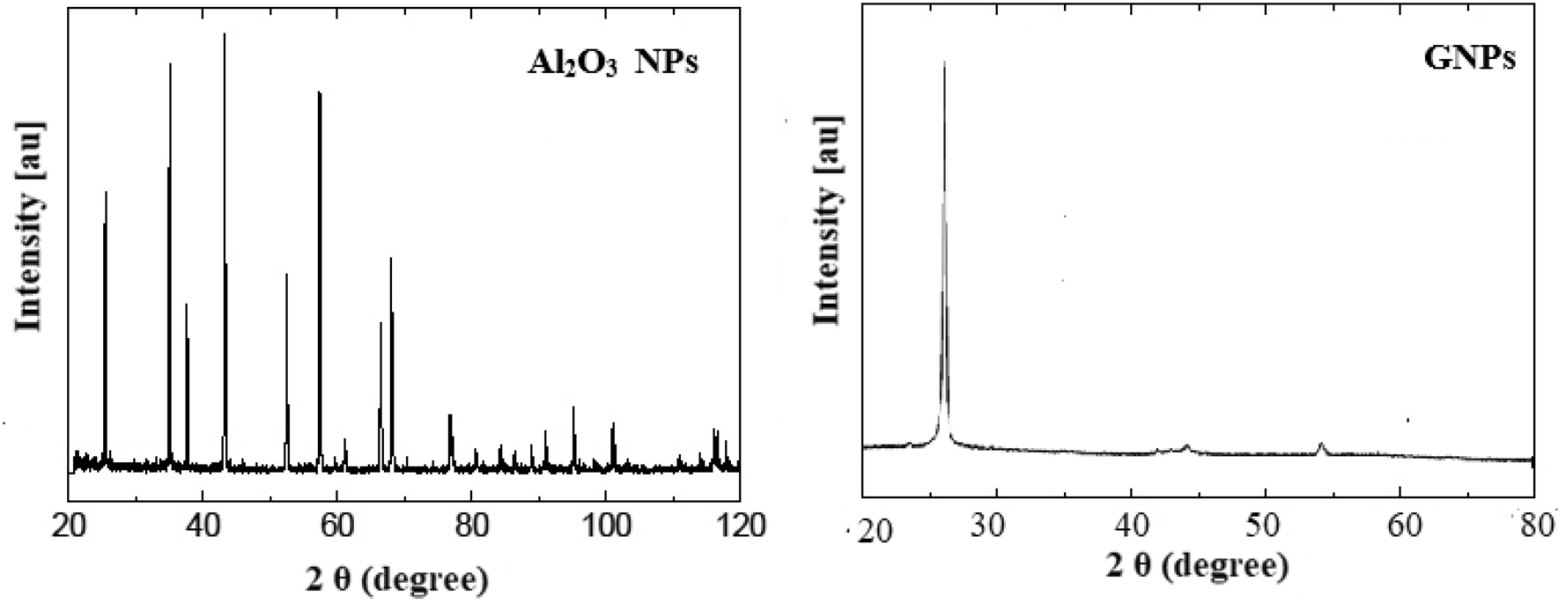
XRD patterns of Al_2_O_3_ NPs and GNPs^36^.

The base material adopted in this current work is High-Density Polyethylene which was purchased from Sigma Aldrich Co. HDPE was a light gray powder with a particle size of 40:90 μm and a density of 0.94 gm/cm^3^. However, Al_2_O_3_ NPs and graphene nanoplatelets (GNPs) were added as hybrid nanofillers which were supplied by US Research Nanoparticles, Inc. Table 1 displays the properties of nanofillers. SEM topography and XRD analysis for Al_2_O_3_ NPs and nanographene were illustrated in Figs. 1 and 2, respectively. The SEM topography showed Al_2_O_3_ NPs as spherical particles while the GNPs were displayed as layers. Moreover, the hybrid of Al_2_O_3_ NPs/GNPs has been confirmed as an ideal filler component to modify the HDPE characteristics. Figure 2 gives the XRD pattern for Al_2_O_3_ NPs and GNPs. It can be indicated that XRD peaks for Al_2_O_3_ NPs were located at diffraction angles (2θ) of 26.5°, 35°, 38.2°, 43.5°, 51.1°, 58.5°, 65.7°, and 78.5°^35^. While XRD analysis for GNPs exhibits a great intensity peak at a diffraction angle of 26.5° and a set of low-intensity peaks at diffraction angles of 42°, 45°, and 56°^32^.

**Table 1:** Technical properties of titanium oxide.

Property	Al_2_O_3_ NPs	GNPs
Purity %	99+	95
Color	white	black
Size (nm)	80	2–8 thickness
Layers	–	3–6
SSA (m^2^/g)	15	500–1000
Bulk Density (gm/cm^3^)	0.42	0.05–0.081

The nanocomposite specimens were prepared using a rotating mixer to obtain a homogeneous dispersion. The HDPE powder was mixed with the nanofiller into a beaker and stirred at 250 rpm for 30 min to incorporate and disperse the nanofiller. The powder mixture is filled in copper molding (10 mm in diameter) under pressure approximately of 15 MPa. The specimens are then exposed to temperatures up to 200 °C for 40 min under a pressure of 25 MPa. The composite is left to cool and then polished. The filler was added with compositions of 0.5%, 1.0%, 1.5%, and 2.0 wt.% of Al_2_O_3_ NPs and 0.5%, and 1.0 wt.% of GNPs. The compositions of HDPE nanocomposite specimens were illustrated in Table 2.

**Table 2:** Different sample compositions of hybrid HDPE nanocomposites.

Sample no	HDPE (%)	Al_2_O_3_ NPs (%)	GNPs (%)
HDPE-00	100	–	–
HDPE-11	99.0	0.5	0.5
HDPE-12	98.5	1.0	0.5
HDPE-13	98.0	1.5	0.5
HDPE-14	97.5	2.0	0.5
HDPE-21	98.5	0.5	1.0
HDPE-22	98.0	1.0	1.0
HDPE-23	97.5	1.5	1.0
HDPE-24	97.0	2.0	1.0

## Experimental setup

### Mechanical properties

The compressive yield strength and modulus of elasticity were evaluated using united high-capacity smart universal hydraulic (DFM-300KN). The specimens were examined based on ASTM standard D1621. However, based on ASTM standard D2240, the hardness results were assessed using the Durometer Shore D. All the specimens were tested 5 times at different positions and the average values were calculated.

### IR analysis

IR spectroscopy was performed to evaluate and identify the characterization of nanocomposite specimens. The test was applied via a spectrophotometer, Beckman IR 4250—USA, and the results were given in approximately 400–2000 cm^−1^ range.

### Tribological properties

**Figure 3:**
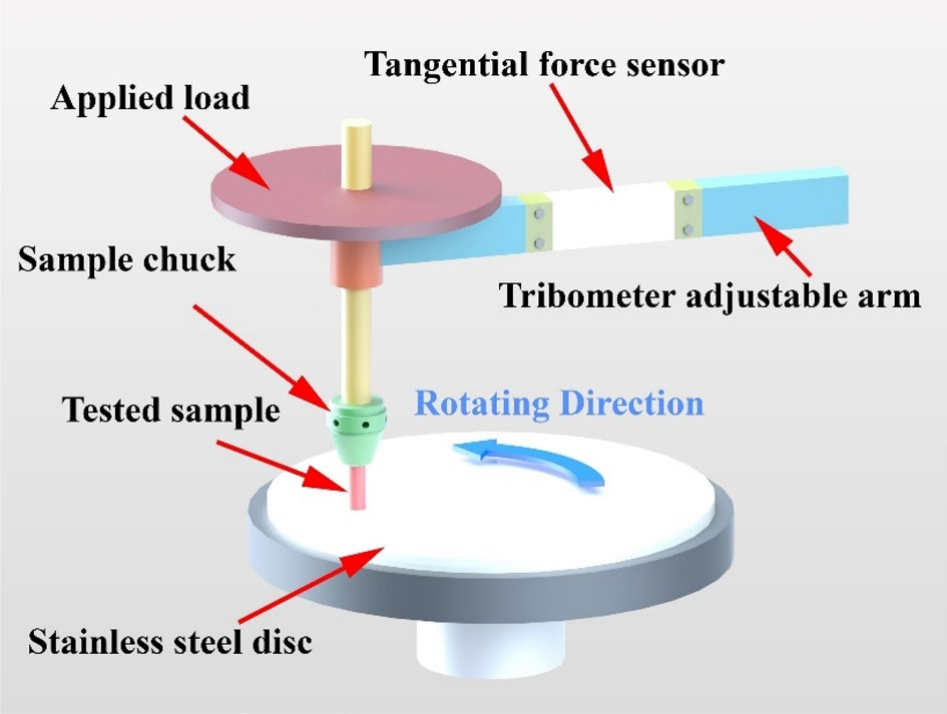
Frictional test rig set-up.

The tribological characteristics of nano-composites specimens were adopted using a pin-on-disc tribometer, as illustrated in Fig. 3. Nano-composite and pure HDPE specimens were installed into the holder and stainless-steel alloy plates were used as counter-face surfaces. A surface roughness tester was utilized to precisely gauge the accurate roughness ofhe stainless-steel plate (Surface roughness of R_a_ = 0.023 μm, R_q_ = 0.029 μm, and R_z_ = 0.179 μm). Specimens have been tested at the room temperature of 35 °C and relative humidity of 60% under dry sliding conditions. The software is provided to take the friction coefficient data and plot the charts of the tested specimens. Experiments were conducted under five different amounts of the normal load of 2, 4, 6, 8, and 10 N at 0.1 m/s of sliding velocity. The abrasive paper with a grit of 1200, according to Standard ANSI grit^37^, was used as a counter-face to examine the wear behavior of nano-composite specimens. Wear tests were done each exactly 120 s., and the weight loss was considered by weighing the specimens before and after the test. The wear rate was determined as follows:

$${\text{W}}_{{\text{r}}} = \Delta {\text{m}}/{\text{L}}\rho {\text{F}}_{{\text{n}}}$$

where the sliding distance L, material density ρ, and applied load F_n_, and proportional to the weight loss Δm.

After the wear test, an electronic microscope (OLYMPUS BX53M, USA) was utilized to analyze the topography of the worn surfaces. The images were confirmed into 2D and 3D to evaluate the wear resistance of the specimens. For more details, Scanning the surfaces via SEM microscope (JCM-6000Plus; JEOL, Tokyo, Japan) was used. While the specimens need a sequence of procedures. So, the specimens were washed and after that dried using an air bath, then the specimens were coated with a thin layer of platinum to contribute to taking a clear SEM shoot.

## Results and discussion

### Mechanical properties

**Figure 4:**
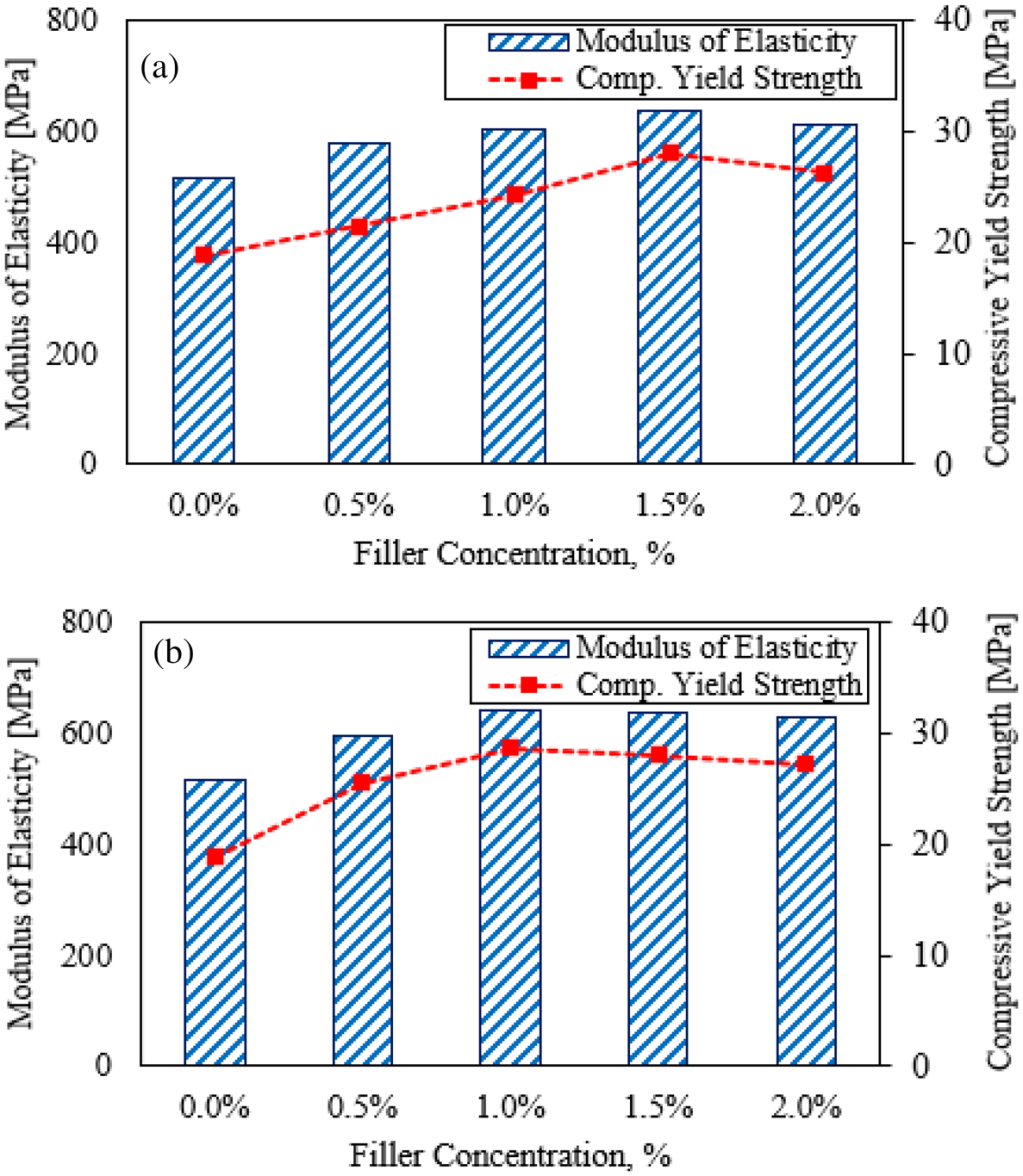
Modulus of Elasticity and compressive yield strength of HDPE/Al_2_O_3_ nanocomposites with loading content of GNPs NPs (**a**) 0.5 wt.% and (**b**) 1.0 wt.%.

**Figure 5:**
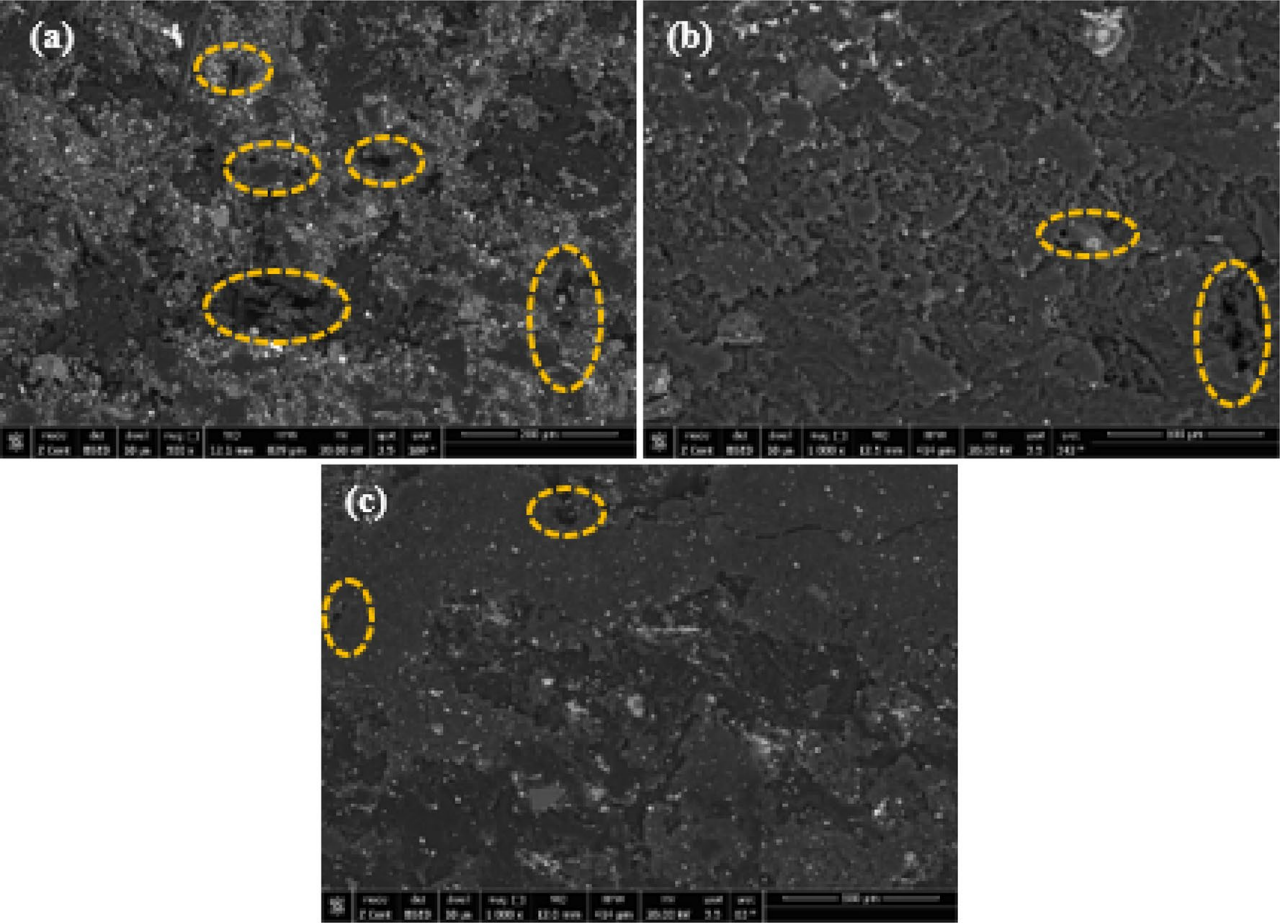
SEM images of composites surfaces: (**a**) HDPE 00, (**b**) HDPE 13 and (**c**) HDPE 21.

**Figure 6:**
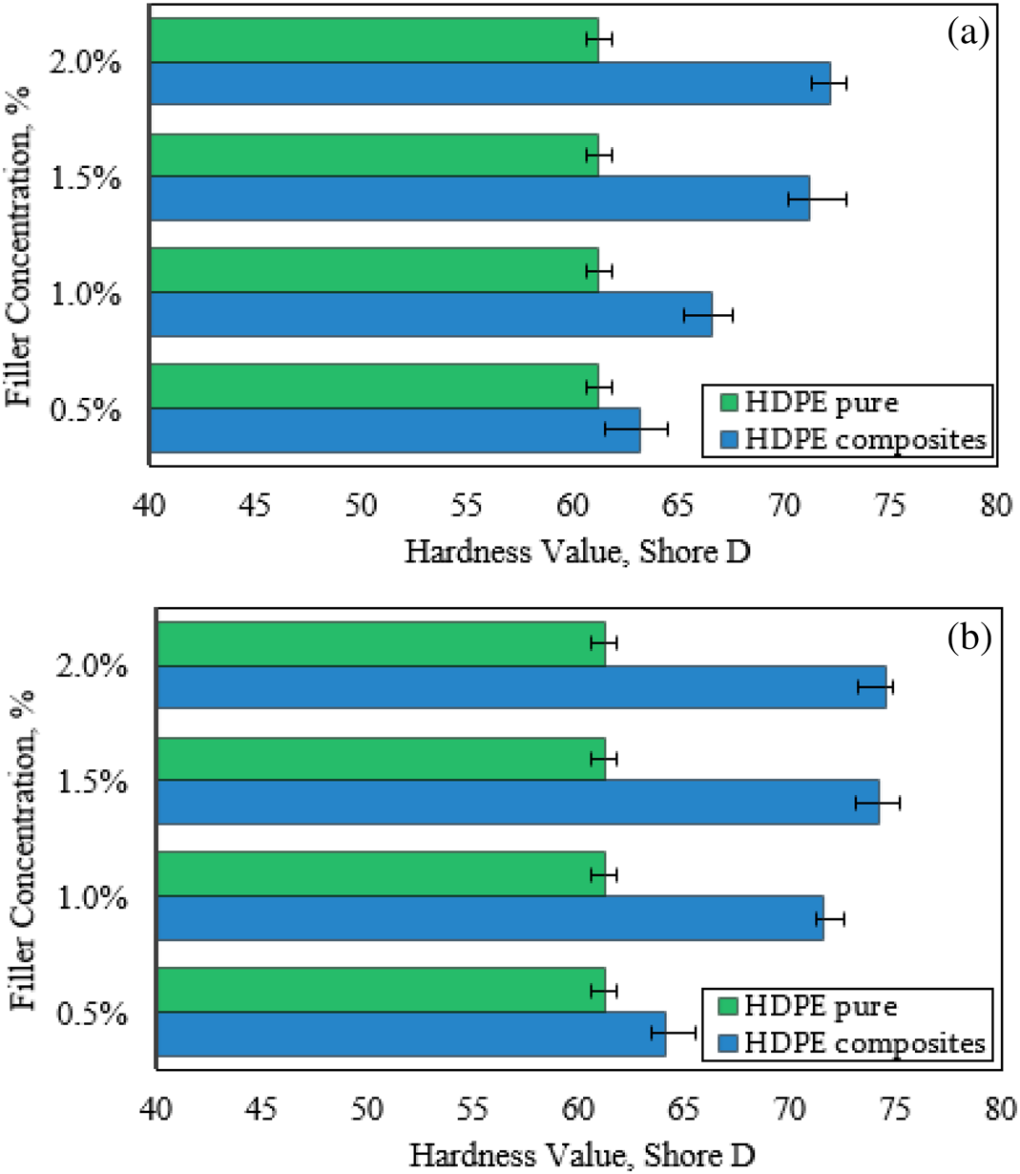
Hardness values of HDPE/Al_2_O_3_ nanocomposites with loading content of GNPs NPs (**a**) 0.5 wt.% and (**b**) 1.0 wt.%.

This current study focused on assessing the mechanical and wear resistance characteristics of HDPE specimens loading with a hybrid nanofiller of Al_2_O_3_ NPs and GNPs. The results were adopted to evaluate the favorable loading content of the hybrid nanofiller. The specimens were divided into two sets, the first was reinforced with 0.5 wt.% of GNPs and the second was performed by adding 1.0 wt.% of GNPs. The influence of nanofiller loading content on the mechanical properties of HDPE composites was illustrated in Figs. 4 and 6. Whereas, the modulus of elasticity, compressive yield strength, and hardness values were presented. The results could be attributed that the dispersion of hybrid nanofiller Al_2_O_3_ NPs and GNPs contributing to improving the mechanical properties of HDPE nanocomposites. The enhancement of mechanical properties may be attributed to the Al_2_O_3_/GNPs which contributes to strengthening the matrix^38,39^. It can be observed from Fig. 4a that the modulus of elasticity and compressive yield improves with increasing of loading amount up to 1.5 wt.% of Al_2_O_3_ NPs and 0.5 wt.% of GNPs. Moreover, the modulus of elasticity and compressive yield strength exhibits a distinct enhancement of about 23.4 and 48%, respectively. Only beyond that limit, it is clear that the properties of the HDPE nanocomposites are taken to deteriorate. Figure 4b illustrates the relationship between the loading amount and the modulus of elasticity and compressive yield strength. Generally, it can be indicated that the specimens containing 1.0 wt.% of GNPs exhibit a better reaction compared to the previous set. This can be attributed to the increase of GNPs loading content plays a vital role to reduce the aggregation of NPs which leads to enhancing the bonding between nanofillers and the HDPE matrix^40^. The maximum improvement value was achieved with a specimen reinforced by 1.0 wt.% of Al_2_O_3_ NPs and 1.0 wt.% of GNPs. The modulus of elasticity and compressive yield strength was enhanced up to 24% and 51.8%, respectively, in comparison with the pure HDPE. One of the most important reasons affecting the strength of the HDPE matrix, which cannot be overlooked, is porosity. It can be evident that the strength of the HDPE matrix decreases due to the presence of porosity, which reduces their load-bearing capability^41,42^. However, in this study, the use of a hybrid filler with a higher surface area restricts the matrix porosity and improves the distribution of the HDPE matrix. SEM images of three HDPE nanocomposite specimens were illustrated in Fig. 5. SEM analysis of pure HDPE matrix, Fig. 5a, revealed a surface morphology with furrows and pores. It can be observed that nanocomposite specimens revealed similar smooth surface morphology and confirmed the absence of the micro-pores on a smooth HDPE surface, as displayed in Fig. 5b and c. This can be attributed to the presence of Al_2_O_3_/GNPs loading content leads to an even distribution of hybrid Al_2_O_3_/GNPs in nano-enriched HDPE composites and restrict the matrix porosity.

While the hardness values of the HDPE nanocomposites were displayed in Fig. 6. Generally, the dispersion of the hybrid nanofiller leads to an increase in the hardness of the HDPE nanocomposites. This could be attributed to increasing nanofiller content contributing to strength filler-matrix bonding, which consequently raises the hardness^31^. It can be indicated that the specimen reinforced by 2.0 wt.% of Al_2_O_3_ NPs and 0.5 wt.% of GNPs has a hardness of 72.1 Shore D, with 17.8% higher than the pure specimen. Moreover, the hardness of the specimen reinforced by 2.0 wt.% of Al_2_O_3_ NPs and 1.0 wt.% of GNPs was found to be 74.6 Shore D, about increasing of 22.8%. According to the previously presented results, there is no doubt that the hybrid nanofiller Al_2_O_3_/GNPs is considered a distinct filling material for the HDPE matrix, which is consistent with previous results^43^.

### IR spectra analysis

**Figure 7:**
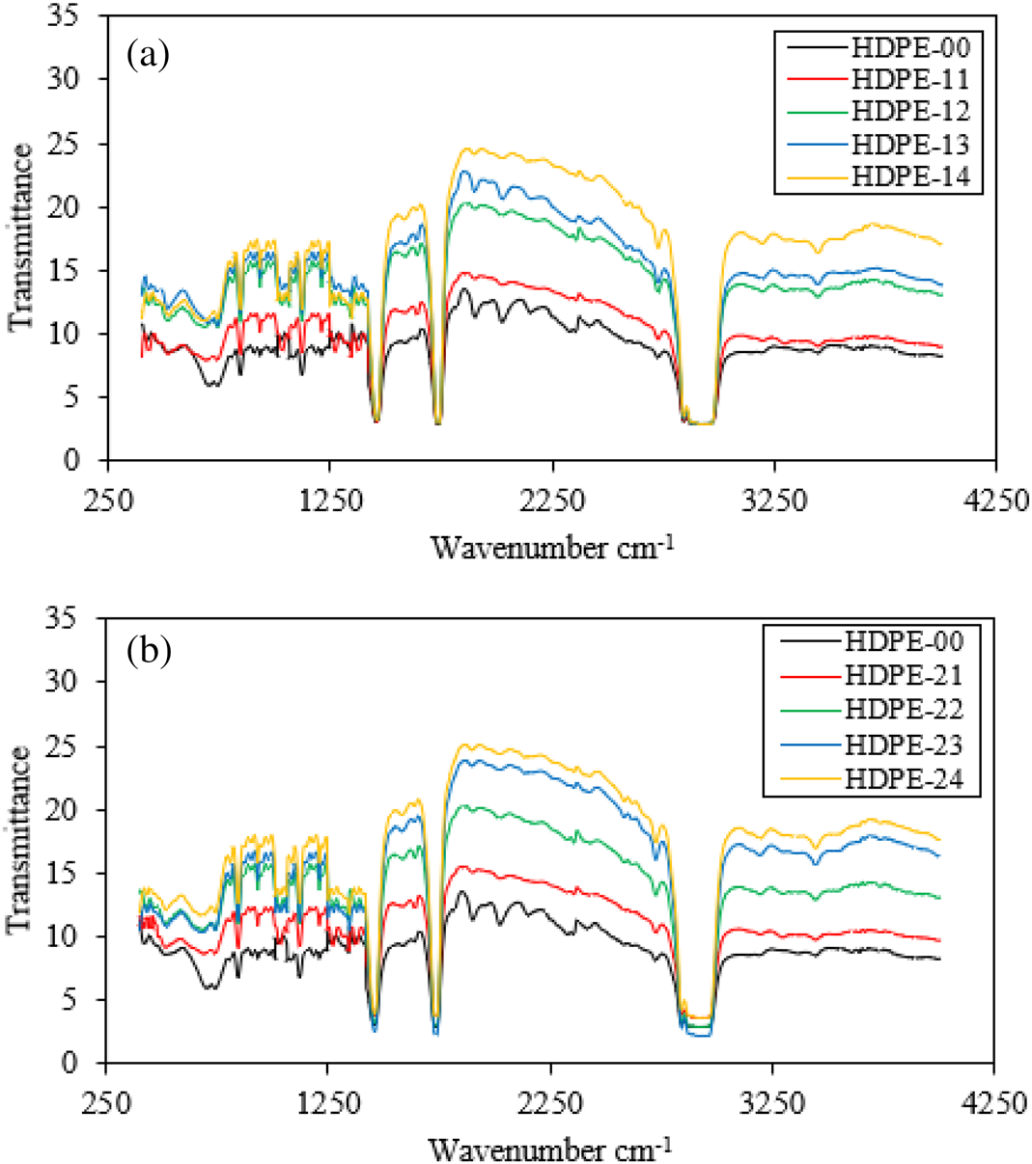
IR spectra analysis of HDPE/Al_2_O_3_ nanocomposites with loading content of GNPs NPs (**a**) 0.5 wt.% and (**b**) 1.0 wt.%.

IR spectra analysis was performed to evaluate the bonding reactions between the filler and the matrix. The chemical bonding of pure HDPE and its composites was displayed in Fig. 7. For the pure HDPE spectra, it can be assigned the many positions on its IR band are starched around wave numbers as 732, 1461, 2873, and 2965 cm^−1^. This may be due to the features of the polyethylene spectrum, which contains fluctuation deformation, symmetric and asymmetric stretching, and bending modes. Moreover, the fluctuation defamation of methylene groups appears at 732 cm^−1^, bending deformation of methylene groups performs at 1461 cm^−1^, and symmetric and asymmetric stretching bonding of methylene groups appear at bands of 2873 to 2965 cm^−1^^44,45^. IR spectra analysis for HDPE nanocomposites exhibits a similar spectrum band to HDPE pure. The presence of fillers through the HDPE matrix leads to an increase in the intensity of the spectrum compared with the pure specimen. These results reflect a good reaction to matching the filler with the HDPE matrix.

### Tribological performance

**Figure 8:**
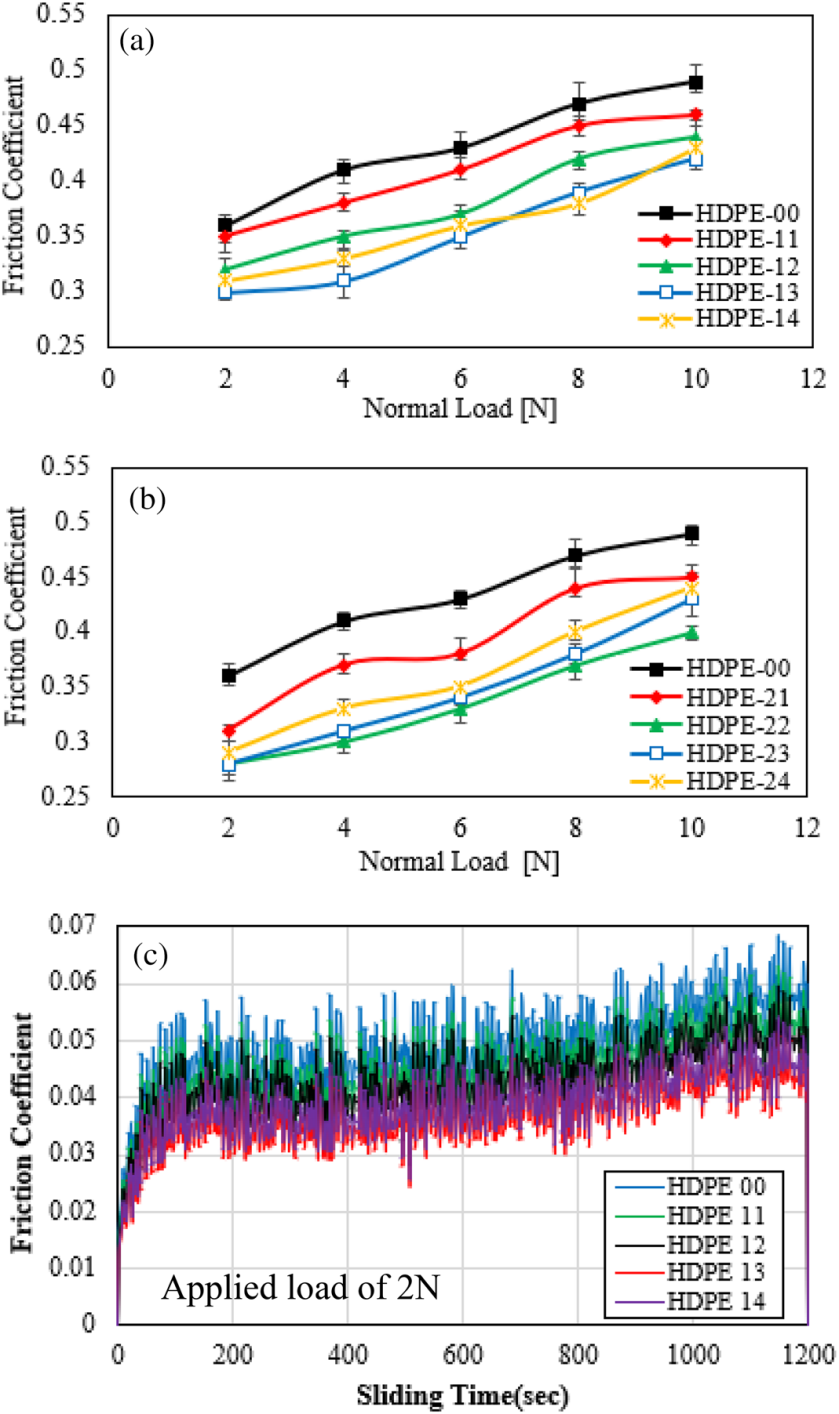
Friction coefficient of HDPE/Al_2_O_3_ nanocomposites with loading content of GNPs (**a**) 0.5 wt.%; (**b**) 1.0 wt.%; (**c**) real-time friction graph at an applied load of 2 N.

The tribological performance was evaluated by examining the coefficient of friction and wear rate of the HDPE nanocomposites. Moreover, the worn surface examinations were performed to identify the wear mechanism. The main concept of this section is aimed to specify the suitable loading amount that has the best tribological performance. The relation between friction coefficient and Al_2_O_3_/GNPs nanofiller content was illustrated in Fig. 8. It can be noticed that the friction coefficient is clearly reduced by adding the hybrid nanofiller with different loading contents. In all the loading cases, the tribological properties of the HDPE nanocomposites were lower than the pure specimen. For specimens filled with 0.5 wt.% of GNPs, Fig. 8a, the COF gradually decreases due to increasing loading content up to 1.5 wt.% of Al_2_O_3_ NPs, and then a reverse reaction occurs. This clearly indicates poor bonding between the filler and the matrix leading to an undesirable tribological performance. The HDPE nanocomposite specimen of 1.5 wt.% of Al_2_O_3_ NPs exhibited the best COF with a reduction of 13% in comparison with the pure specimen. This may be because the hybrid nanofiller contributes to the unique characteristics of both substances. The rolling effect of the Al_2_O_3_ NPs and the self-lubrication of GNPs play a direct role in reducing the friction in the contact area^6,9^. Figure 8b illustrates the influence of the increased loading content of GNPs to 1.0 wt.% on the COF. Generally, the HDPE specimens dispersed by 1.0 wt.% of GNPs exhibit lower COF in comparison with the other set. This may be due to the spread of graphene on the surface of the nanocomposite^46,47^. The lowest COF was achieved with the specimen with contents of 0.5 wt.% of Al_2_O_3_ NPs and 1.0 wt.% of GNPs, as the reduction ratio reached 23%. It can be obvious that the high loading content of Al_2_O_3_ NPs leads to increase COF, this may be due to agglomeration within the matrix^34^. Meanwhile, all HDPE composites exhibited apparently the same stick–slip behavior as shown in Fig. 8c.

**Figure 9:**
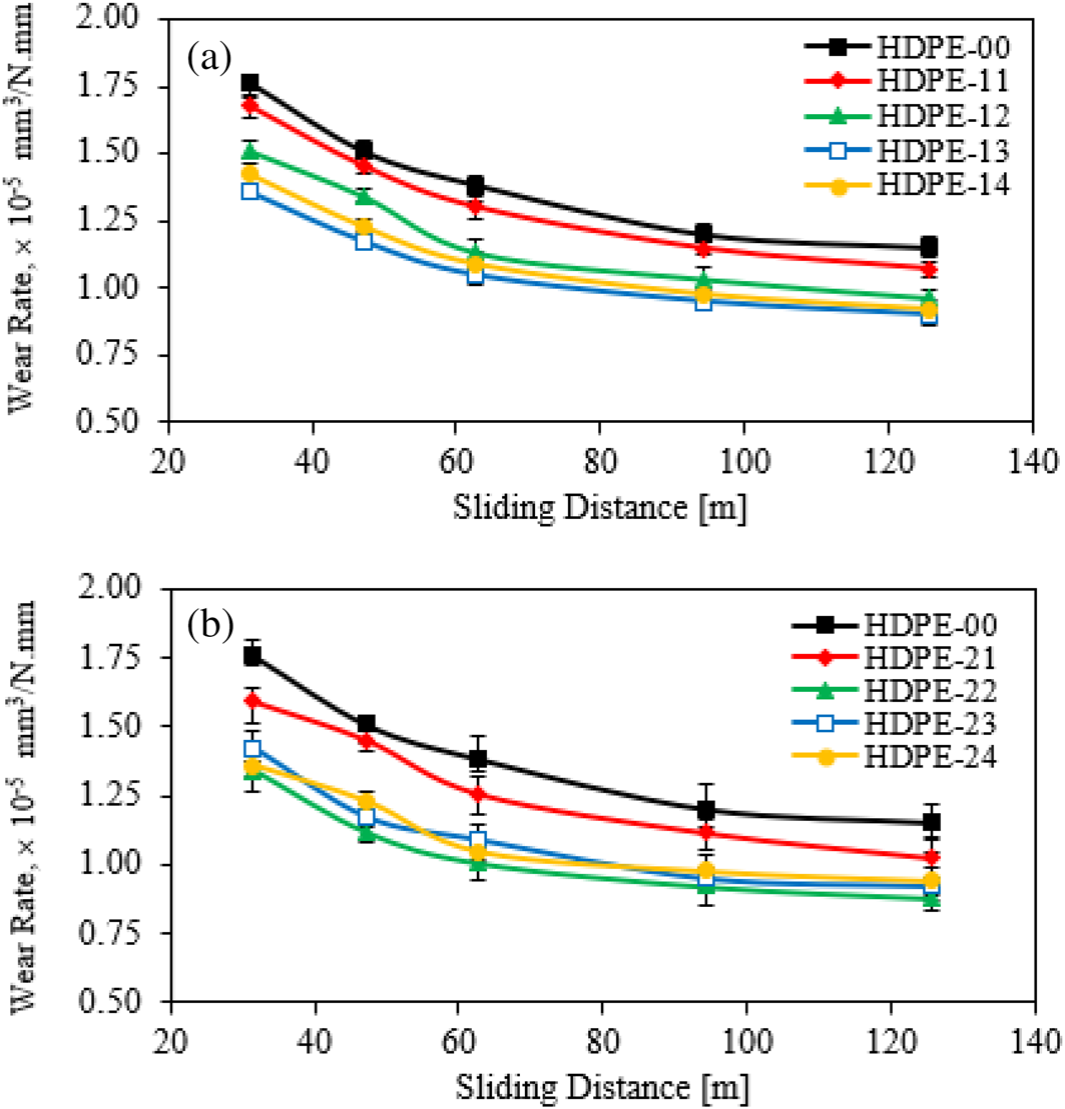
Wear rate of HDPE/Al_2_O_3_ nanocomposites with loading content of GNPs NPs (**a**) 0.5 wt.% and (**b**) 1.0 wt.%.

The wear rate was estimated versus different sliding distances under an applied load of 8 N, dry condition lubrication, 30 °C temperature, and relative humidity of 60%. The wear rate of HDPE specimens reinforced with loading content of GNPs of 0.5 and 1.0 wt.% was given in Fig. 9a and b, respectively. The same trend of COF is repeated with the study of wear rate for HDPE nanocomposites. The specimens filled with a hybrid nanofiller exhibit a lower wear rate than the pure specimen^31^. Figure 9a indicated that the minimum wear rate can be obtained when 1.5 wt.% of Al_2_O_3_ NPs was added to the HDPE nanocomposite, it may be due to the graphene nanoplatelets making and improving the self-lubricating behavior. The wear rate lessened by 19% less than the pure specimen. On the other side, the wear rate specimens reinforced with 1.0 wt.% of GNPs were displayed in Fig. 9b. Therefore, it can be observed that the HDPE nanocomposite of 1.0 wt.% of Al_2_O_3_ NPs exhibited a wear rate 26% lower in the comparison with the free additives specimen. Finally, the specimen filled with 2.0 wt.% of hybrid Al_2_O_3_ NPs/GNPs, with a ratio of 1:1, has a favorable wear resistance among the all-loading content. Thus, the self-lubricating effect of graphene contributes to improving the tribological performance of HDPE nanocomposites.

**Figure 10:**
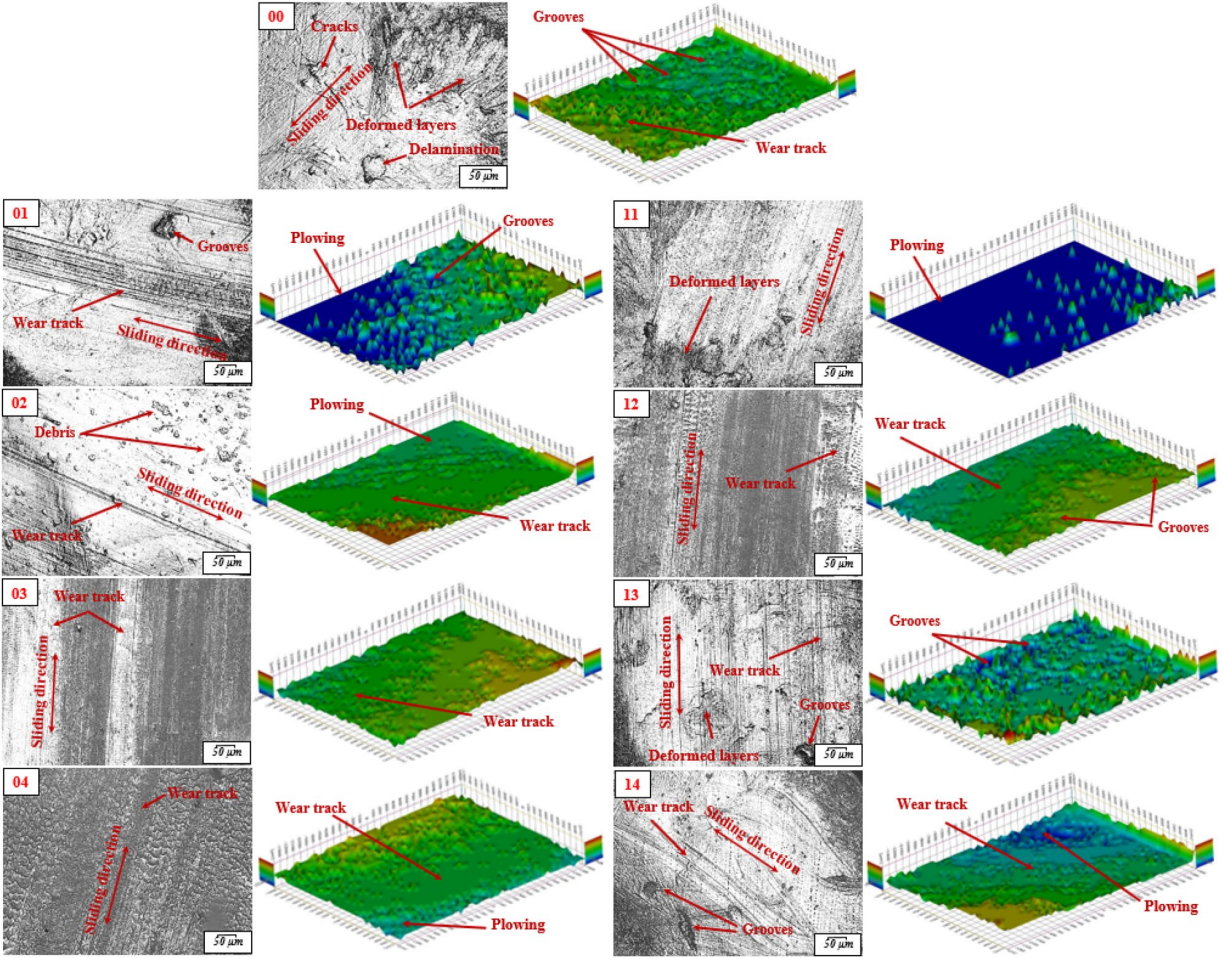
Optical images and 3D topography of worn surfaces of HDPE/Al_2_O_3_/GNPs nanocomposites.

**Figure 11:**
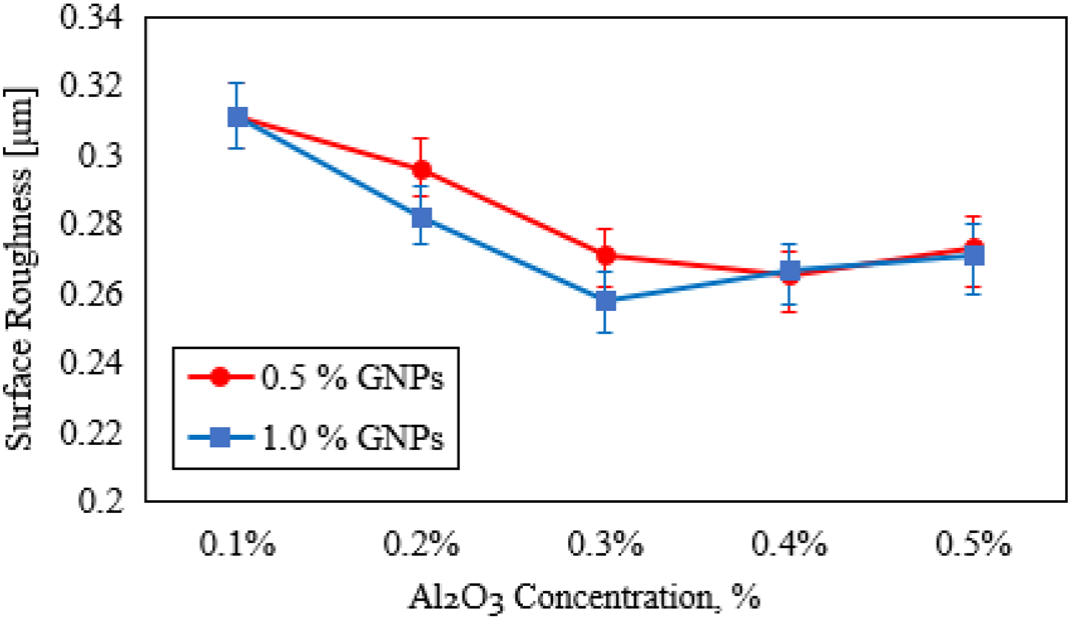
Surface roughness of worn surfaces of HDPE/Al_2_O_3_/GNPs nanocomposites.

To further clarify the wear mechanism, the worn surfaces of the specimens were examined. Optical images, 3D topography, and SEM of worn surfaces were scanned to evaluate and analyze the frictional performance of HDPE specimens. Figure 10 displays the 2D and 3D topography images. The worn surface of the specimen (00), free of fillers, cavities, and cracks appear on the wear track. While those defects are reduced and the surfaces of the specimens improve as a result of adding hybrid filler to the mixture, which enhanced the wear resistance^48^. It is possible to note that specimen (10) has a clear plowing and wear track in the contact area. The worn surfaces of the specimens continue to improve the wear rates as the depth of the wear track decreases as indicated in the specimen (02). It is obvious that specimen (03) has a good surface roughness and low deformation surface, which is evidence of the improvement of the strength and hardness of the nanocomposite. In contrast, specimen (04) shows plowing on the surface and a clear wear track. From the above, it can conclude that the specimen achieves the best tribological performance compared to this set of specimens. About the second set of specimens filled with 1.0 wt.% GNPs, it can notice that specimen (12) has a smoother worn surface and no deformed layers. Nanocomposites which contain 2.0 wt.% of Al_2_O_3_ NPs and GNPs with an equal ratio showed the most favorable effect. Figure 11 displays the surface roughness of specimens. It can be observed the pure specimen has the highest surface roughness. The results revealed that loading of hybrid nanofiller leads to reducing the surface roughness^49,50^. It was demonstrated that the specimen with a loading content of 1.0 wt.% of Al_2_O_3_ NPs and GNPs exhibit the lowest surface roughness and smoother. It may be due to a uniform distribution of nanofiller through the HDPE matrix that contributes to enhancing the strength of resin and restricting voids and furrows.

**Figure 12:**
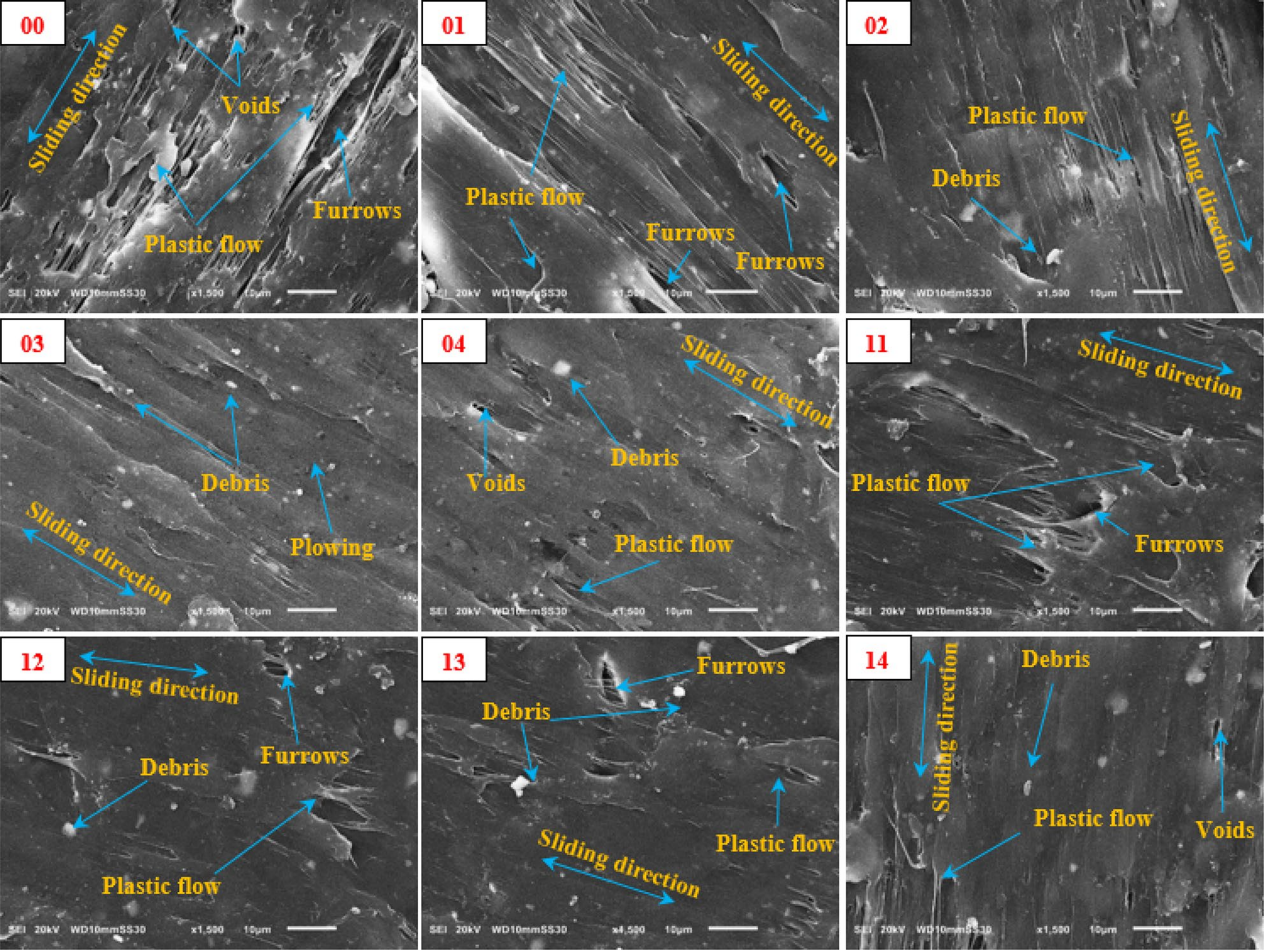
SEM images of worn surfaces of HDPE/Al_2_O_3_/GNPs nanocomposites.

To give more detailed overviews of the wear marks, the worn surfaces were examined by SEM images of specimens, as displayed in Fig. 12. The pure specimen view shows obvious damage to the surface. Nonetheless, many voids, plastic deformation flow, and furrows at the same direction of sliding appear on the worn surface. It was also observed that specimens reinforced with a hybrid nanofiller exhibit fewer plastic layers and cracks. These plastic flows are not caused by friction with the disc but are produced as a result of the presence of debris on the contact area. Therefore, the debris size is an active factor in the deformation of the surface. Moreover, the GNPs form a self-lubricating layer that contributes to reducing the frictional force^51^. Based on that, specimen (03) exhibits a good wear resistance surface, and the wear debris is smaller. This could be attributed to the hybrid nanofiller being compatible with the HDPE matrix, which contributes to modifying the characteristics of the nanocomposites. The same observations are true for the other specimens set. However, the plastic layers, cracks, and furrows are less than the pure specimen. The worn surfaces displayed a smoother and less damaged area, this refers to the good bonding between filler and matrix.

## Conclusions

The current study focused on the contributions of hybrid nanofiller Al_2_O_3_ NPs and GNPs on the HDPE mechanical and tribological features. Two sets of specimens with loading content of 0.5% and 1.0 wt.% of GNPs were adopted. Based on the experimental results, the conclusions can be drawn:

1. IR spectra analysis proved a distinct dispersion of the Al_2_O_3_ NPs and GNPs additive in the matrix without any problems between the bonds.
2. The mechanical properties of HDPE nanocomposites were enhanced, where the modulus of elasticity and compressive yield stress was raised to 23.4 and 48%, respectively, at contents of 1.5 wt.% of Al_2_O_3_ NPs and 0.5 wt.% of GNPs.
3. Specimen filled by 1.0 wt.% of Al_2_O_3_ NPs and 1.0 wt.% of GNPs, displays better mechanical characteristics, so the modulus of elasticity and compressive yield stress achieved an improvement up to 24 and 51.8%, respectively.
4. High-loading content leads to recording the maximum hardness values, where the hardness improves by 22.8%.
5. The hybrid nanofiller contributes to reducing the friction coefficient by 13%, for HDPE contains 1.5 wt.% of Al_2_O_3_ NPs and 0.5 wt.% of GNPs. Meanwhile, 1.0 wt.% of Al_2_O_3_ NPs and 1.0 wt.% of GNPs HDPE composites showed a friction coefficient 23% less than Pure HDPE.
6. The minimum wear rate exhibited with specimen filled by 1.0 wt.% of Al_2_O_3_ NPs and 1.0 wt.% of GNPs, where the reduction is about 26%.
7. Loading content of 1.0 wt.% of Al_2_O_3_ NPs and 1.0 wt.% of GNPs exhibits the best mechanical and tribological performance.

## Data Availability

The datasets used and/or analysed during the current study available from the corresponding author on reasonable request.

## References

[1] Goswami TK, Mangaraj S (2011). Advances in polymeric materials for modified atmosphere packaging (MAP). Multifunctional and Nanoreinforced Polymers for Food Packaging.

[2] Xu S, Tangpong XW (2013). Tribological behavior of polyethylene-based nanocomposites. J. Mater. Sci..

[3] Suh NP, Mosleh M, Arinez J (1998). Tribology of polyethylene homocomposites. Wear.

[4] Sahebian S, Zebarjad SM, Sajjadi SA, Sherafat Z, Lazzeri A (2007). Effect of both uncoated and coated calcium carbonate on fracture toughness of HDPE/CaCO_3_ nanocomposites. J. Appl. Polym. Sci..

[5] Nabhan, A., Ameer, A. K. & Rashed, A. Tribological and mechanical properties of HDPE reinforced by Al_2_O_3_ nanoparticles for bearing materials (2019).

[6] Guermazi N, Elleuch K, Ayedi HF, Fridrici V, Kapsa P (2009). Tribological behaviour of pipe coating in dry sliding contact with steel. Mater. Des..

[7] Anderson JC (1982). High density and ultra-high molecular weight polyethenes: Their wear properties and bearing applications. Tribol. Int..

[8] Kurtz SM (2009). UHMWPE Biomaterials Handbook: Ultra High Molecular Weight Polyethylene in Total Joint Replacement and Medical Devices.

[9] Baena JC, Wu J, Peng Z (2015). Wear performance of UHMWPE and reinforced UHMWPE composites in arthroplasty applications: A review. Lubricants.

[10] Taha M, Hassan M, Dewidare M, Kamel MA, Ali WY, Dufresne A (2021). Evaluation of eco-friendly cellulose and lignocellulose nanofibers from rice straw using Multiple Quality Index. Egypt. J. Chem..

[11] Havelin LI (2009). The Nordic Arthroplasty Register Association: A unique collaboration between 3 national hip arthroplasty registries with 280,201 THRs. Acta Orthop..

[12] Shahemi N, Liza S, Abbas AA, Merican AM (2018). Long-term wear failure analysis of uhmwpe acetabular cup in total hip replacement. J. Mech. Behav. Biomed. Mater..

[13] Pakhaliuk VI, Vasilets VN, Poliakov AM, Torkhov NA (2022). Reducing the wear of the UHMWPE used in the total hip replacement after low-pressure plasma treatment. J. Appl. Comput. Mech..

[14] Oonishi H, Kuno M, Tsuji E, Fujisawa A (1997). The optimum dose of gamma radiation–heavy doses to low wear polyethylene in total hip prostheses. J. Mater. Sci. Mater. Med..

[15] Mahboba, Y. J. & Al-Shammari, M. A. Enhancing wear rate of high-density polyethylene (HDPE) by adding ceramic particles to propose an option for artificial hip joint liner. In *IOP Conference Series: Materials Science and Engineering* Vol. 561 12071 (2019).

[16] Rashed A, Nabhan A (2018). Influence of adding nano graphene and hybrid SiO_2_-TiO_2_ nano particles on tribological characteristics of polymethyl methacrylate (PMMA). KGK Kautsch. Gummi Kunstst..

[17] Elshemy EA, Showaib EA (2020). Effect of filler loading on erosive characteristics of epoxy/SiO_2_ coatings. Solid State Technol..

[18] Pelto J, Heino V, Karttunen M, Rytöluoto I, Ronkainen H (2020). Tribological performance of high density polyethylene (HDPE) composites with low nanofiller loading. Wear.

[19] Sahu SK, Badgayan ND, Samanta S, Sreekanth PSR (2020). Experimental investigation on multidimensional carbon nanofiller reinforcement in HDPE: An evaluation of mechanical performance. Mater. Today Proc..

[20] Xue Y, Wu W, Jacobs O, Schädel B (2006). Tribological behaviour of UHMWPE/HDPE blends reinforced with multi-wall carbon nanotubes. Polym. Test..

[21] Ferreira FV (2016). Correlation of surface treatment, dispersion and mechanical properties of HDPE/CNT nanocomposites. Appl. Surf. Sci..

[22] Ferreira EHC, Vieira AA, Vieira L, Fechine GJM (2021). High-tribological-performance polymer nanocomposites: An approach based on the superlubricity state of the graphene oxide agglomerates. Polymers.

[23] Naveen, G. J. *et al.* Role of Graphene oxide and addition of MoS_2_ in HDPE matrix for improved tribological properties. In *IOP Conference Series: Materials Science and Engineering* Vol. 376 12077 (2018).

[24] Bahrami H, Ramazani A, Shafiee M, Kheradmand A (2016). Preparation and investigation of tribological properties of ultra-high molecular weight polyethylene (UHMWPE)/graphene oxide. Polym. Adv. Technol..

[25] Gallab M, Taha M, Rashed A, Nabhan A (2022). Effect of low content of Al_2_O_3_ nanoparticles on the mechanical and tribological properties of polymethyl methacrylate as a denture base material. Egypt. J. Chem..

[26] Xiong DS, Lin JM, Fan DL (2006). Wear properties of nano-Al_2_O_3_/UHMWPE composites irradiated by gamma ray against a CoCrMo alloy. Biomed. Mater..

[27] Panin, S. V., Kornienko, L. A., Alexenko, V. O., Buslovich, D. G. & Dontsov, Y. V. Extrudable polymer-polymer composites based on ultra-high molecular weight polyethylene. In *AIP Conference Proceedings* Vol. 1915 20005 (2017).

[28] Hwang HJ, Jung SL, Cho KH, Kim YJ, Jang H (2010). Tribological performance of brake friction materials containing carbon nanotubes. Wear.

[29] Pöllänen M, Pirinen S, Suvanto M, Pakkanen TT (2011). Influence of carbon nanotube–polymeric compatibilizer masterbatches on morphological, thermal, mechanical, and tribological properties of polyethylene. Compos. Sci. Technol..

[30] Sahu, S. K., Badgayan, N. D. & Sreekanth, P. S. R. Understanding the influence of contact pressure on the wear performance of HDPE/multi-dimensional carbon filler based hybrid polymer nanocomposites. *Wear* **438** (2019).

[31] Dabees S, Tirth V, Mohamed A, Kamel BM (2021). Wear performance and mechanical properties of MWCNT/HDPE nanocomposites for gearing applications. J. Mater. Res. Technol..

[32] Wang Q (2017). Preparation of high antistatic HDPE/polyaniline encapsulated graphene nanoplatelet composites by solution blending. RSC Adv..

[33] Xu, S. *et al.* Wear and friction of carbon nanofiber-reinforced HDPE composites (2012).

[34] Di Maro M, Duraccio D, Malucelli G, Faga MG (2021). High density polyethylene composites containing alumina-toughened zirconia particles: Mechanical and tribological behavior. Compos. Part B Eng..

[35] Badi N, Mekala R, Khasim S, Roy AS, Ignatiev A (2018). Enhanced dielectric performance in PVDF/Al-Al_2_O_3_ core–shell nanocomposites. J. Mater. Sci. Mater. Electron..

[36] Aldrich, S. Sigma Aldrich (2019).

[37] Zipperian D (2002). Silicon carbide abrasive grinding. Qual. Matters Newsl..

[38] Colom X, Carrasco F, Pages P, Canavate J (2003). Effects of different treatments on the interface of HDPE/lignocellulosic fiber composites. Compos. Sci. Technol..

[39] Kanagaraj S, Varanda FR, Zhil’tsova TV, Oliveira MSA, Simões JAO (2007). Mechanical properties of high density polyethylene/carbon nanotube composites. Compos. Sci. Technol..

[40] Saba N, Md Tahir P, Jawaid M (2014). A review on potentiality of nano filler/natural fiber filled polymer hybrid composites. Polymers.

[41] Kumar S, Ramesh MR, Doddamani M, Rangappa SM, Siengchin S (2022). Mechanical characterization of 3D printed MWCNTs/HDPE nanocomposites. Polym. Test..

[42] Mozumder MS, Mourad A-HI, Mairpady A, Pervez H, Haque ME (2018). Effect of TiO_2_ nanofiller concentration on the mechanical, thermal and biological properties of HDPE/TiO_2_ nanocomposites. J. Mater. Eng. Perform..

[43] Dabees S, Kamel BM, Tirth V, Elshalakny AB (2020). Experimental design of Al_2_O_3_/MWCNT/HDPE hybrid nanocomposites for hip joint replacement. Bioengineered.

[44] Maheswari CU, Reddy KO, Muzenda E, Shukla M, Rajulu AV (2013). A comparative study of modified and unmodified high-density polyethylene/borassus fiber composites. Int. J. Polym. Anal. Charact..

[45] Yang L, Zhang F, Endo T, Hirotsu T (2003). Microstructure of maleic anhydride grafted polyethylene by high-resolution solution-state NMR and FTIR spectroscopy. Macromolecules.

[46] Liu T, Li B, Lively B, Eyler A, Zhong W-H (2014). Enhanced wear resistance of high-density polyethylene composites reinforced by organosilane-graphitic nanoplatelets. Wear.

[47] Nabhan A, Taha M, Ghazaly NM (2023). Filler loading effect of Al_2_O_3_/TiO_2_ nanoparticles on physical and mechanical characteristics of dental base composite (PMMA). Polym. Test..

[48] Pelto J (2019). Matrix morphology and the particle dispersion in HDPE nanocomposites with enhanced wear resistance. Polym. Test..

[49] Topcu FT, Erdemir U, Sahinkesen G, Yildiz E, Uslan I, Acikel C (2010). Evaluation of microhardness, surface roughness, and wear behavior of different types of resin composites polymerized with two different light sources. J. Biomed. Mater. Res. Part B Appl. Biomater..

[50] Nabhan A, Sherif G, Abouzeid R, Taha M (2023). Mechanical and tribological performance of HDPE matrix reinforced by hybrid Gr/TiO2 NPs for hip joint replacement. J. Funct. Biomater..

[51] Omrani E, Moghadam AD, Kasar AK, Rohatgi P, Menezes PL (2021). Tribological performance of Graphite nanoplatelets reinforced Al and Al/Al_2_O_3_ self-lubricating composites. Materials.

